# Diagnostic value of chest computed tomography imaging for COVID-19 based on reverse transcription-polymerase chain reaction: a meta-analysis

**DOI:** 10.1186/s40249-021-00910-8

**Published:** 2021-10-21

**Authors:** Jing Liu, Xue Yang, Yunxian Zhu, Yi Zhu, Jingzhe Liu, Xiantao Zeng, Hongjun Li

**Affiliations:** 1 Department of Radiology, The Affiliated Infectious Diseases Hospital of Soochow University, The Fifth People’s Hospital of Suzhou, Suzhou, 215000 Jiangsu People’s Republic of China; 2 Department of Radiology, Beijing Youan Hospital Capital Medical University, Beijing, 100069 People’s Republic of China; 3 Department of Radiology, The First Hospital of Tsinghua University, Beijing, 100016 People’s Republic of China; 4 Center for Evidence-Based and Translational Medicine, Zhongnan Hospital of Wuhan University, Wuhan, 430071 Hubei People’s Republic of China

**Keywords:** COVID-19, Tomography, X-ray computed, Reference standard

## Abstract

**Background:**

The computed tomography (CT) diagnostic value of COVID-19 is controversial. We summarized the value of chest CT in the diagnosis of COVID-19 through a meta-analysis based on the reference standard.

**Methods:**

All Chinese and English studies related to the diagnostic value of CT for COVID-19 across multiple publication platforms, was searched for and collected. Studies quality evaluation and plotting the risk of bias were estimated. A heterogeneity test and meta-analysis, including plotting sensitivity (Sen), specificity (Spe) forest plots, pooled positive likelihood ratio (+LR), negative likelihood ratio (-LR), dignostic odds ratio (DOR) values and 95% confidence interval (*CI*), were estimated. If there was a threshold effect, summary receiver operating characteristic curves (SROC) was further plotted. Pooled area under the receiver operating characteristic curve (AUROC) and 95% *CI* were also calculated.

**Results:**

Twenty diagnostic studies that represented a total of 9004 patients were included from 20 pieces of literatures after assessing all the aggregated studies. The reason for heterogeneity was caused by the threshold effect, so the AUROC = 0.91 (95% *CI*: 0.89–0.94) for chest CT of COVID-19. Pooled sensitivity, specificity, +LR, -LR from 20 studies were 0.91 (95% *CI:* 0.88–0.94), 0.71 (95% *CI*: 0.59–0.80), 3.1(95% *CI:* 2.2–4.4), 0.12 (95% *CI:* 0.09–0.17), separately. The *I*^*2*^ was 85.6% (*P* = 0.001) by Q-test.

**Conclusions:**

The results of this study showed that CT diagnosis of COVID-19 was close to the reference standard. The diagnostic value of chest CT may be further enhanced if there is a unified COVID-19 diagnostic standard. However, please pay attention to rational use of CT.

**Graphic Abstract:**

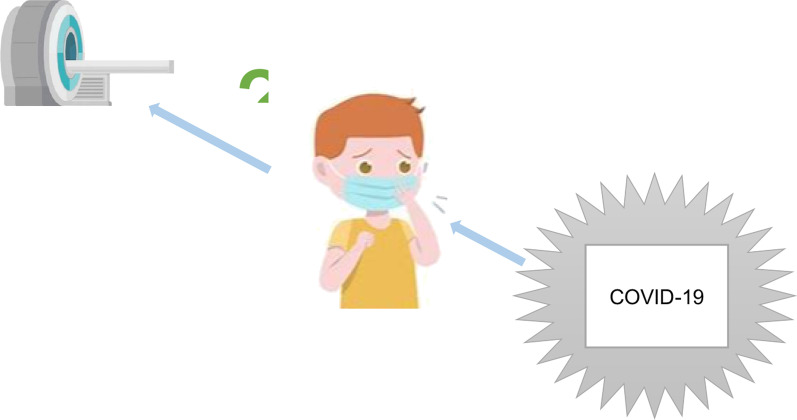

## Background

Coronavirus disease 2019 (COVID-19) is an acute infectious disease caused by severe acute respiratory syndrome coronavirus 2 (SARS-CoV-2) and is primarily characterized by pulmonary inflammatory lesions. It can also cause damage to the digestive system [1–3], nervous system [4, 5], urinary system [6] and other systemic organs, with corresponding clinical symptoms. COVID-19 was identified as the health emergency of national concern by World Health Organization on 30 January, 2020 since its initial detection in December 2019 [7]. COVID-19 has highly transmissible [8]. Current clinical studies show that there are no specific therapeutic drugs for treatment [9].

The reference standard for diagnosing COVID-19 is reverse transcription-polymerase chain reaction (RT-PCR) [10]; however, it suffers from numerous flaws, such as poor sensitivity [11], producing false negatives, etc., all of which have also been noted in the studies [12, 13]. A study indicated that RT-PCR was not a perfect reference standard [14]. What’s more, a succession of countries [15] had reported the discovery of COVID-19 variant strains, and the proportion is increasing. The most feared of the COVID-19 variants could accelerate the spread of the disease. Researchers at Vita’s laboratory in Helsinki, Finland considered that RT-PCR may not be able to detect the mutated virus (http://www.zhihu.com/question/445217928). Feng et al. pointed out that the abundance of genomic data made it possible to reassess the applicability of RT-PCR to ensure that it was applicable to mutant strain [16].

Computed tomography (CT) played an important role in the global fight against COVID-19 [11]. Some studies have shown that CT can be used as a primary auxiliary screening and diagnostic tool in pandemic areas [17–19]. The COVID-19 pandemic has put enormous pressure on global health resources, which creates an urgent need for researchers to find a reasonable diagnosis. In this meta-analysis, we aim to summarize the diagnostic value and existing problems of CT.

## Methods

### Search strategy

We searched for published studies in English and Chinese as well as what could be considered gray area studies in PubMed, Embase, Web of Science, Cochrane Library, CBM (http://sinomed.ac.cn/), CNKI (https://www.cnki.net/), Wan-fang (https://www.wanfangdata.com.cn/index.html), and VIP (http://cstj.cqvip.com/) databases between 1 December, 2019 and 16 August, 2021. The search strategy was developed under the guidance of professionals and involved matching subject terms with free words. The PubMed search strategy was (((“COVID-19”[Mesh]) OR (((((((((((2019 novel coronavirus disease[Title/Abstract]) OR (COVID19[Title/Abstract])) OR (COVID-19 pandemic[Title/Abstract])) OR (SARS-CoV-2 infection[Title/Abstract])) OR (COVID-19 virus disease[Title/Abstract])) OR (2019 novel coronavirus infection[Title/Abstract])) OR (2019-nCoV infection[Title/Abstract])) OR (coronavirus disease 2019[Title/Abstract])) OR (coronavirus disease-19[Title/Abstract])) OR (2019-nCoV disease[Title/Abstract])) OR (COVID-19 virus infection[Title/Abstract]))) AND ((“Tomography, X-Ray Computed”[Mesh]) OR ((((((X-Ray Comput* Tomography[Title/Abstract]) OR (CT[Title/Abstract])) OR (comput* tomograph*[Title/Abstract])) OR (CT Scan*[Title/Abstract])) OR (Helical CT*[Title/Abstract])) OR (CT X Ray*[Title/Abstract])))) AND (sensitiv*[Title/Abstract] OR sensitivity and specificity[MeSH Terms] OR (predictive[Title/Abstract] AND value*[Title/Abstract]) OR predictive value of tests[MeSH Term] OR accuracy*[Title/Abstract]). We prospectively submitted the meta-analysis protocol for registration of PROSPERO (CRD42020225262).

### Selection criteria

Inclusion criterias were as follow-up: (1) retrospective or prospective diagnostic test study; (2) collect clinical history, suspected COVID-19, confirmed COVID-19, and chest CT results of diagnosed/suspected COVID-19; (3) COVID-19 confirmed by the reference standard RT-PCR; (4) direct or indirect information sufficient to extract 2 × 2 table data for diagnosis of COVID-19 by chest CT, including true positive (TP), false positive (FP), false negative (FN) and true negative (TN).

Literatures were excluded if they were (1) duplicates, case reports, reviews, systematic reviews, meta-analyses, conference abstracts, letters, non-original studies, and unrelated studies; (2) failure to extract the 2 × 2 table data; (3) the full text was unavailable; (4) not confirmed by the reference standard.

### Data extraction and quality assessment

Two authors independently screened the studies for quality assessment and data extraction according to the inclusion and exclusion criteria, and disagreements were resolved by reaching a consensus or consulting a third senior specialist. The extracted data included: (1) First author, study site, study time, publication year, age, gender, sample size, study type (prospective, retrospective), and case selection; (2) Diagnostic reference standard, i.e., RT-PCR; (3) Outcome indicators included the sensitivity (Sen), specificity (Spe), accuracy (Ac), negative predictive value (NPV), positive predictive value (PPV), TP, FP, FN, and TN of diagnosis by chest CT, which were used to build the 2 × 2 table.

A quality evaluation was done in the diagnostic test of Review Manager 5.3 (the Cochrane Collaboration, London, UK), and for the requirements of Quality Assessment of Diagnostic Studies-2 (QUADAS-2), the evaluation rules applicable to this study were developed by referring to relevant studies [20]. On this basis, if the opinions of the two authors were still not consistent, the evaluation rules would be renegotiated to reach a consensus and further improve and supplement the pre-established evaluation rules.

### Statistical analysis

Meta-disc1.4.0.0 (Clinical Biostatistics Unit, Madrid, Spain) and Stata14.0 (Computer Resource Center, Texas, US) software were used for statistical analysis. The Q test was utilized to analyze the heterogeneity between the results of each study. If *P* < 0.01, the difference was considered statistically significant, and the heterogeneity was quantitatively judged based on *I*^*2*^ value. If *I*^*2*^ > 50%, heterogeneity was considered significant, and the random effects model was selected. Otherwise, the fixed effects model was selected; Spearman’s rank correlation coefficient and summary receiver operating characteristic curve (SROC) between the logarithm of sensitivity and the logarithm of (1 − specificity) were used to determine whether there was a threshold effect. Forest plots of Sen and Spe were made to calculate the pooled positive likelihood ratio (+LR), negative likelihood ratio (-LR), dignostic odds ratio (DOR) values and 95% confidence interval (*CI*); if there was a threshold effect, SROC was further plotted to calculate the pooled area under the receiver operating characteristic curve (AUROC). Publication bias was assessed with Deeks' plots.

## Results

### Literature search

**Fig. 1:**
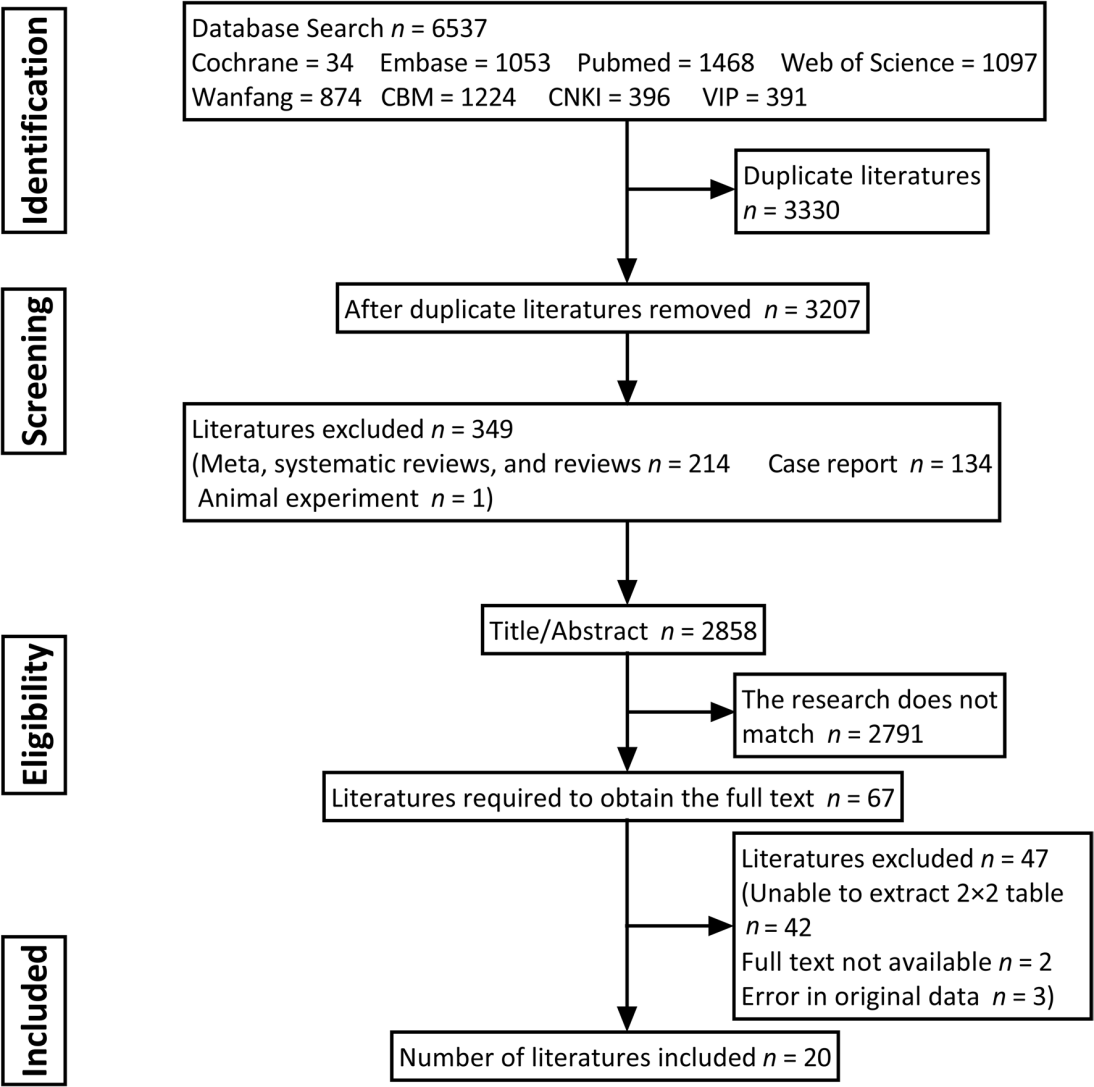
Flowchart of literature selection process used for this meta-analysis. CBM (http://sinomed.ac.cn/), CNKI (https://www.cnki.net/), Wan-fang (https://www.wanfangdata.com.cn/index.html), and VIP (http://cstj.cqvip.com/)

The flow chart for the screening of included literatures is shown in Fig. 1. According to the search strategy, a total of 6537 pieces of literatures were initially screened; 3330 duplicates were excluded by the management application; 214 pieces of literatures of meta-analysis, systematic review and review were excluded; 134 pieces of literatures of case report were excluded; 1 piece of literature of animal experiment was excluded. In addition, 2791 pieces of literatures with irrelevant content were excluded after the titles and abstracts were reviewed; 42 pieces of literatures from which the 2 × 2 table could not be obtained were excluded after a review of the full text; 3 pieces of literatures were error in original data; 2 pieces of literatures without full text. Finally, 20 pieces of literatures were included, which were from China, Italy, France, United Kingdom, the Netherlands, Brazil and India.

### Quality evaluation

The basic characteristics of the included 20 literatures are shown in Table 1. These literatures were produced by emergency departments or fever clinics in different regions.

**Table 1:** Basic characteristics of relevant included literatures

No	Country	Research time	Published year	Age, years	Gender M/F***	TP*	FP*	FN*	TN*	References
1	China	6 Jan–6 Feb, 2020	2020	51 ± 15	467/547	580	308	21	105	[21]
2	Brazil	Feb–Mar, 2020	2020	19–88	NA**	16	10	9	56	[22]
3	Italy	27 Feb–4 Jul, 2020	2020	59 ± 15.8	288/408	520	61	31	84	[23]
4	Italy	4 Mar–19 Mar, 2020	2020	57 ± 17	83/75	60	42	2	54	[24]
5	China	25 Jan–2 Feb, 2020	2020	51.3 ± 17.1	290/297	423	71	10	83	[25]
6	French	3 Mar–4 Apr, 2020	2020	66.4 ± 18.6	363/331	259	49	28	358	[26]
7	Italy	3 Mar–9 Apr, 2020	2020	62.4 ± 18.2	424/349	419	66	43	245	[27]
8	Netherlands	13 Mar–24 Mar, 2020	2020	66 (55–76)	113/80	74	35	9	75	[28]
9	Brazil	1 Mar–14 Apr, 2020	2020	57.9 ± 18.0	88/71	76	4	10	69	[29]
10	France	1 Mar–28 Mar, 2020	2020	36–75	NA**	105	8	24	77	[30]
11	Netherlands	27 Mar–20 Apr, 2020	2020	NA**	157/162	120	22	13	164	[31]
12	China	13 Jan–31 Jan, 2020	2021	36.9 ± 14.5	113/67	30	15	4	131	[32]
13	China	20 Jan–10 Feb, 2020	2020	50.7 ± 17.1	49/71	88	16	3	13	[33]
14	United Kingdom	23 Mar–15 May, 2020	2020	NA**	NA**	40	37	29	101	[34]
15	France	6 Mar–22 Apr, 2020	2020	NA**	NA**	919	148	172	955	[35]
16	India	15 Feb–30 Jun, 2020	2021	62(44–78)	214/134	206	37	11	94	[36]
17	France	18 Mar–24 Apr, 2020	2021	69 ± 20	257/230	69	60	10	348	[37]
18	China	20 Jan–5 Mar, 2020	2020	21–83	87/77	73	70	3	18	[38]
19	China	1 Feb–7 Feb, 2020	2020	59.4 ± 0.9	105/137	132	56	15	39	[39]
20	China	20 Jan–15 Feb, 2020	2020	8–82	96/68	75	55	4	30	[40]

*TP true positive, *FP* false positive, *FN* false negative, *TN* true negative; **NA not available; ***M = male, F = female

**Fig. 2:**
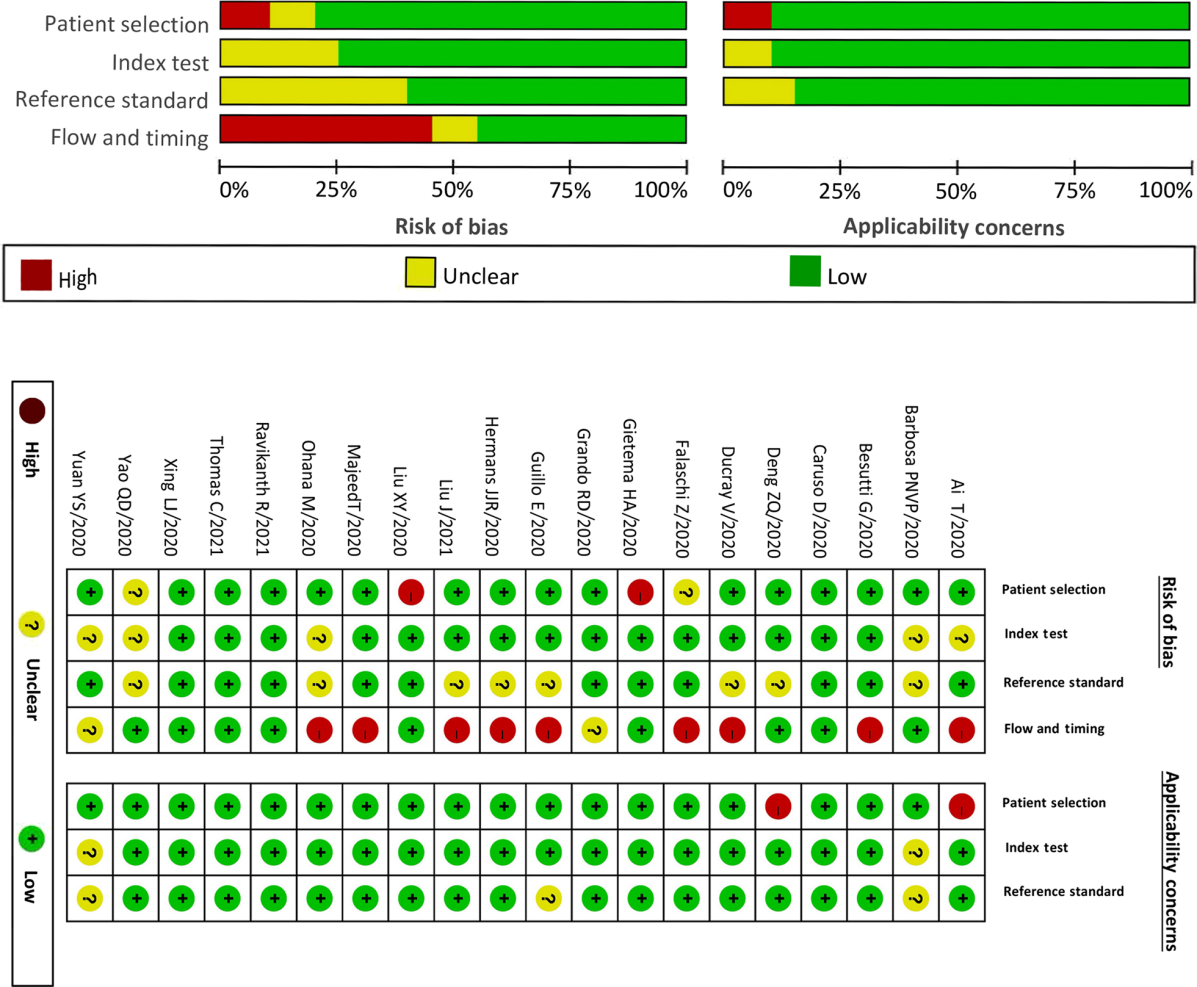
Risk of bias and applicability concerns graph, risk of bias and applicability concerns summary of 20 included literatures using the Quality Assessment of Diagnostic Accuracy Studies-2 (QUADAS-2) tool

The results of QUADAS-2 are shown in Fig. 2. In 9/20 pieces of literatures, the flow and timing were evaluated as high risk because these studies did not include all the cases, which might cause bias. In 8/20 pieces of literatures, the risk of bias with regards to the reference standard was unclear because these studies did not specifically describe whether repeat testing was performed for the first negative RT-PCR test. In 5/20 pieces of literatures, the risk of the index test was unclear because the literatures did not specifically show whether the film was read through the blind method. In 2/20 pieces of literatures, the high risk of patient selection was mainly because all the patients in the studies could be definitively diagnosed by the index test and the lack of patients that were difficult to diagnose. Furthermore, in 2/20 pieces of literatures, the risk of patient selection was unclear because of the absence of explanatory notes confirming the presence of difficult to diagnose patients.

### Pooling of effect sizes and plotting

**Fig. 3:**
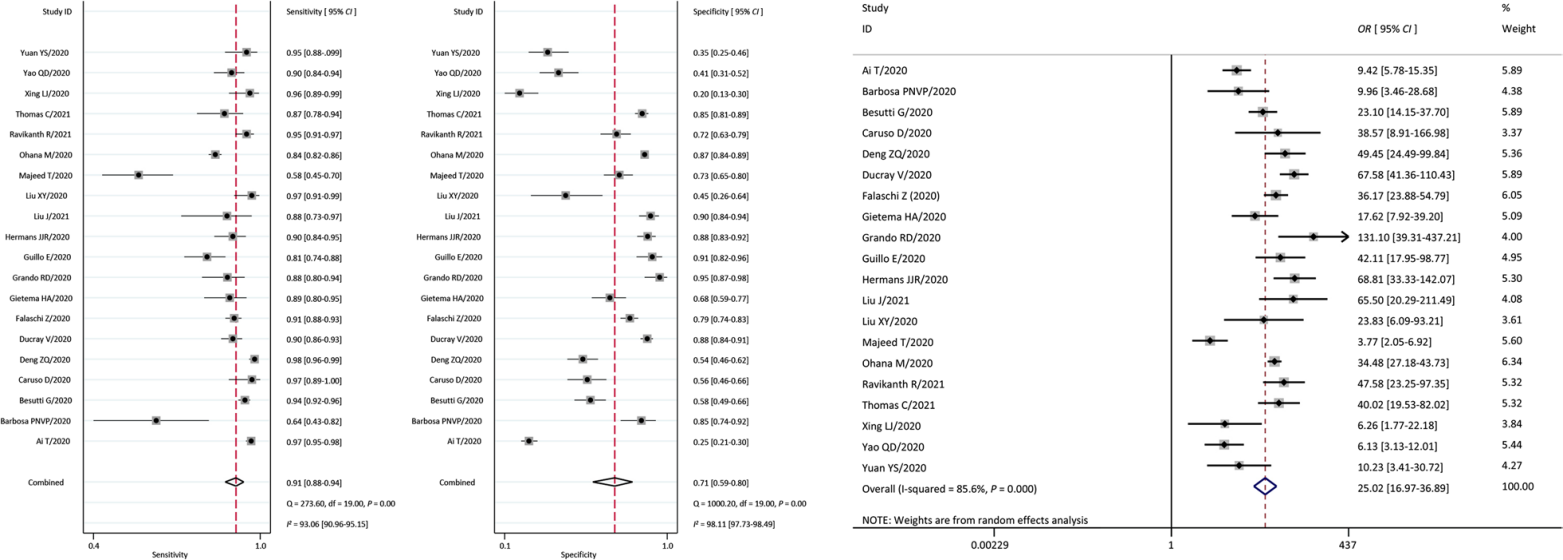
Coupled forest plots of pooled sensitivity, specificity and *OR* of Chest CT. Numbers were pooled with 95% *CI*. Corresponding heterogeneity statistics were provided at the bottom. *OR* odds ratio, *CI* confidence interval

**Fig. 4:**
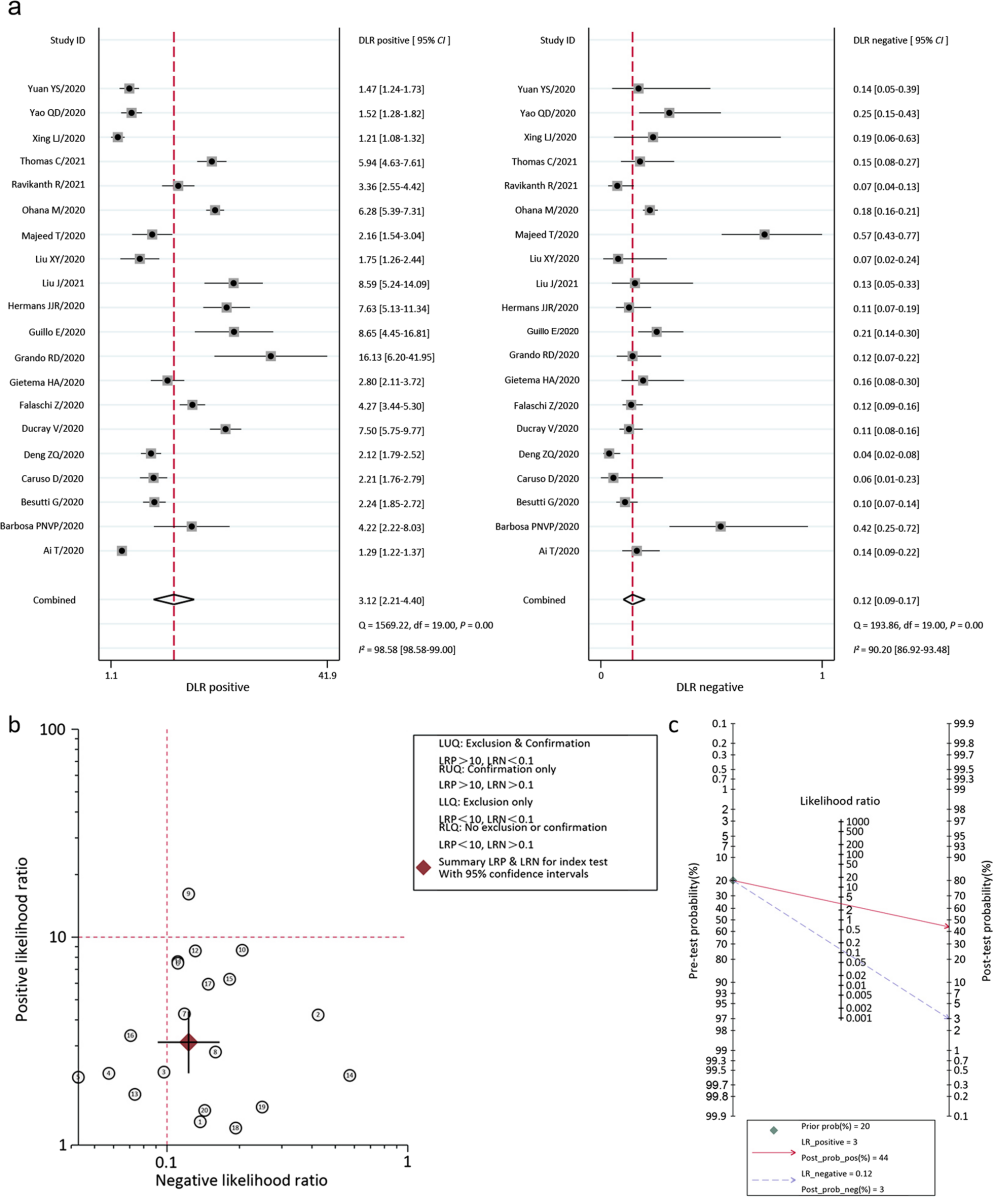
Coupled forest plots of pooled LR positive and LR negative of chest CT. Numbers were pooled with 95% *CI* (**a**). A likelihood ratio matrix diagram which summary LR positive and LR negative for index test with 95% *CI* (**b**)*. The* probability graph shows the relationship between pre-test probability and post-test probability (**c**). *DLR* diagnostic likelihood ratio, *CI* confidence interval, *LR* likelihood ratio, *CT* computed tomography, *LRP* likelihood ratio positive, *LRN* likelihood ratio negative, *RUQ* right upper quadrant, *LLQ* lift lower quadrant, *RLQ* right lower quadrant

Pooled sensitivity, specificity, +LR, -LR from 20 studies were 0.91 (95% *CI:* 0.88–0.94), 0.71 (95% *CI:* 0.59–0.80), 3.1 (95% *CI:* 2.2–4.4), 0.12 (95% *CI:* 0.09–0.17), separately. The *I*^*2*^ was 85.6% (*P* = 0.001) by Q-test. The effect size was pooled with the random-effects model. DOR was 25.02 (95% *CI*: 16.97–36.89). The forest plots of Sen, Spe and DOR (OR) value of CT in the 20 literatures are shown in Fig. 3. The positive likelihood ratio (+LR) and negative likelihood ratio (-LR), the likelihood ratio scatter plot, the pre-test probability/post-test probability is shown in Fig. 4. It can be seen that the misdiagnosis rate and missed diagnosis rate of the index test for the included literatures used for this study are small. In this study, the pre-test probability by the physician was 20%, the post-test positive predictive value was 44%, and the post-test negative predictive value was 3%, indicating that the physician’s judgment of the patient is important. This also indicates that if the pre-test probability is increased, then the index test can proficiently predict the disease.

### Threshold effect analysis

**Fig. 5:**
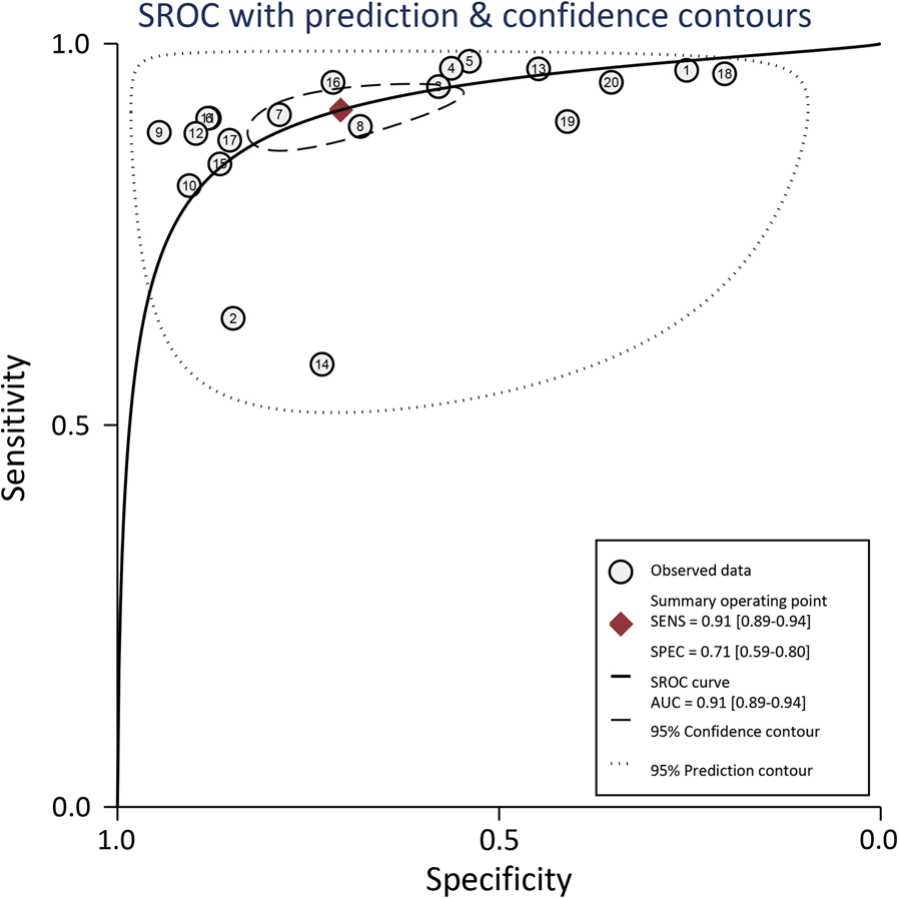
SROC curve indicated that the area under the curve was 0.91, with the 95% *CI.* *SROC* summary receiver operating characteristic curves, *CI* confidence interval, *SENS* sensitivity, *AUC* area under the curve, *SPEC* specificity

The Spearman’s correlation coefficient between the logarithm of sensitivity and the logarithm of (1 − specificity) was determined to be *r* = 0.681 (*P* = 0.001), indicating significant correlation and threshold effect heterogeneity. SROC was plotted, showing a “shoulder-arm”-like change. Therefore, Sen and Spe could not be combined alone in this study. In order to further to reflect the real diagnostic accuracy of CT, AUROC was calculated to be 0.91 (95% *CI*: 0.89–0.94). The results (AUROC > 90%, and *P* < 0.05) indicate that CT has a diagnostic accuracy close to the reference standard, and it is basically capable of making a correct diagnosis. SROC is shown in Fig. 5.

### Sensitivity analysis and Deeks’ publication bias test

**Fig. 6:**
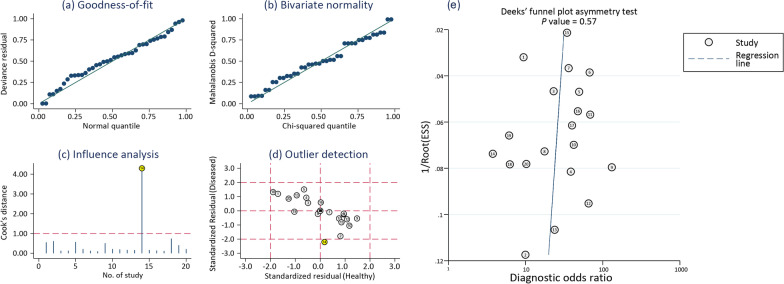
Sensitivity analysis of four aspects included goodness-of-fit, bivariate normality, influence analysis and qutlier detection (**a**–**d**). The publication bias among the included literatures as demonstrated by Deeks’ funnel plot (**e**)

It can be clearly seen from Fig. 6a to d that there is strong sensitivity in one original study (No. 14) [34]. Tracing back to the original study revealed that this study was a prospective cohort study of the diagnosis of COVID-19 in emergency patients at the Department of Emergency Surgery, Royal College of Surgeons, UK, and the cases selected may not have been representative of the general population. Other original studies did not make the results sensitive [21–33, 35–40]. In general, this study features stable results, and AUROC is highly accurate.

It can be clearly seen from the results in Fig. 6e that *P* = 0.57, which indicates that the funnel plot is symmetrical. Therefore, it can be determined that there is no publication bias in this study, and the conclusions of this study are accurate and reliable.

## Discussion

To investigate the value of CT imaging for the diagnosis of COVID-19, 20 diagnostic studies were included in this study. The results prove that the diagnostic value of CT is close to the reference standard. At present, there is disagreement about the role of CT in the diagnosis of COVID-19. Some scholars believed that [18] the chest CT, which served as the primary screening or diagnostic method, had a high false positive rate in areas with low incidences of COVID-19. Others have suggested that [17] chest CT scans could be used as a primary screening and diagnostic tool for COVID-19. COVID-19 Diagnosis and Treatment Protocol (Trial 5th Revised Version) [41] indicated that suspected cases possessing imaging features of pneumonia in Hubei Province as being clinically diagnosed COVID-19. In addition, the progress of COVID-19 is still unclear, and the continuous emergence of mutant strains has puzzled researchers. CT provides macroscopic imaging changes that are not affected by viral mutations, advances in CT thin-slice and reconstruction techniques have increased the detection rate of the disease. At present, asymptomatic infection is increasing and the harm is huge. Chen et al. [42] showed that chest CT was helpful for early detection of asymptomatic infected persons. At the same time, CT provides a visual image representation for the follow-up comparison of diseases. It is still necessary to pay attention to the amount of radiation from CT. Fortunately, it has been reported on the study that the use of isocenter can ensure the same image quality and reduce the radiation of CT [43]. In addition, infection prevention and control management of CT examination rooms is critical.

The results of this study indicate that the heterogeneity is caused by the threshold effect. Combined with the quality evaluation results of QUADAS-2 in this study, there are high risk of bias and applicability concerns in the flow and timing, patient selection. Analyzing the causes of heterogeneity, the authors believe that it depends on the disease prevalence, the characteristics of the study population, diagnostic criteria, etc. In this study, we conducted a CT screening examination of COVID-19 based on the patient population in the emergency department and fever clinic of various hospitals during the epidemic period, which also included some hospitals that isolated and treated suspected patients, so there was a certain patient selection bias. In the included studies, the guidelines or consensus for CT diagnosis of COVID-19 were inconsistent, as were the qualifications and specialty of the diagnosing physician. All these may be the reasons for the threshold effect (heterogeneity) in this study.

The COVID-19 pathogen is affected by various factors and has a low positive detection rate. This indicated that nucleic acid test may produce false negative results [44, 45]. Among the studies, RT-PCR tests have been repeated on patients with negative results, which can reduce the probability of a false negative to a certain extent. We defined positive results and all negative results after the first or multiple RT-PCR tests as COVID-19 positive and COVID-19 negative, respectively. In particularly, multiple RT-PCR tests are necessary for highly suspected patients with negative RT-PCR. The bias of the reference standard was minimized. The main problem at hand for countries with increasing case numbers is the lack of appropriate screening and diagnostic facilities [46]. A recent summary of clinical observations on COVID-19 in China revealed that some patients had positive RT-PCR later than the onset of the clinical symptoms themselves. However, they had CT imaging changes or related clinical symptoms when RT-PCR tests were negative, and due to the failure to produce a timely diagnosis, some patients progressed rapidly, which affected the prognosis.

It has been more than a year since COVID-19 was first confirmed. Further standardization and unification of CT implementation by experts in related fields to improve the pre-test probability of COVID-19 by front-line physicians will benefit doctors and patients worldwide. At the same time, experts on countries need to cooperate in multiple ways. First, a unified diagnostic standard for CT is significant. Up to now, scholars are still exploring and studying. A meta-analysis on evaluating the diagnostic value of CT using methods such as the COVID-19 Reporting and Data System (CO-RADS) and the Radiological Society of North America (RSNA) Classification System [14]. Smet et al. studied the COVID-19 Reporting and Data System (CO-RADS) diagnostic power of CT [47]. China has also published expert consensus on COVID-19 imaging diagnosis [48]. Then, radiologists need to be professionally trained in these guidelines or consensus. Finally, we should pay attention to standardize the visiting procedures for clinically suspected patients and close contacts by screening the epidemiological history and collecting the history of relevant clinical symptoms, such as fever, cough, myalgia or fatigue, dyspnea, expectoration and chest tightness, diarrhea, nausea or vomiting, abdominal discomfort or pain as well as loss of appetite and olfactory dysgeusia [49]. In conclusion, we should conduct nasopharyngeal/oropharyngeal swab tests for suspected patients and CT examinations for RT-PCR-negative patients. Patients suspected of COVID-19 via the initial results of a CT diagnosis should be isolated and the diagnosis should be further confirmed by multiple RT-PCR tests or specifically RT-PCR tests for lower respiratory tract specimens. Antibody results could also be used to provide further confirmation [45].

There are some limitations of this study. Firstly, there are differences in the regional and medical policies of COVID-19, and the studies we included may not represent the characteristics of the general population for the area in which the study was based. Secondly, most of the studies we included are retrospective studies, and prospective study designs have relatively small sample sizes. Thirdly, the initial inexperience of front-line physicians may have affected the diagnostic performance of CT. Fourthly, given that most of the studies are retrospective, we are concerned about the thoroughness of the blind diagnostic results of CT and RT-PCR. Finally, there may be bias in RT-PCR due to the different reference standards, kits used, sampling methods and target genes detected in each country.

## Conclusions

In this study, we mainly summarized the value of CT in diagnosing COVID-19, but it also had some limitations. As a rapid and intuitive means of diagnosing chest disease, CT is recommended for rational use for PCR-negative patients with high suspicion of COVID-19. In the event of a global pandemic, the combination of CT and RT-PCR can quickly and accurately confirm a diagnosis and interrupt the transmission chain.

## Data Availability

The dataset supporting the conclusion of this article is available from the corresponding author upon reasonable request.

## Abbreviations

COVID-19: Coronavirus disease 2019
QUADAS-2: Quality assessment of diagnostic studies-2
RT-PCR: Reverse transcriptase-polymerase chain reaction
*CI*: Confidence interval
DOR: Diagnostic odds ratio
SROC: Summary receiver operating characteristic curve
AUROC: Area under the receiver operating characteristic curve
+LR: Positive likelihood ratio
-LR: Negative likelihood ratio
Sen: Sensitivity
Spe: Specificity
Ac: Accuracy
NPV: Negative predictive value
PPV: Positive predictive value
TP: True positive
FP: False positive
FN: False negative
TN: True negative
CT: Computed tomography
